# Perioperative Advances in Repair of Abdominal Aortic Aneurysm: A Narrative Review of Strategies to Enhance Outcomes and Reduce Complications

**DOI:** 10.7759/cureus.83205

**Published:** 2025-04-29

**Authors:** Malik K Al-Ariki, Abubakar I Sidik, Debraj Ghosh, Md Limon Hossain, Rojina Asadi, Rajiv Mishra, Ahmad M.A. Abuirayyeh, Em Samnang, Mussalim I Kairatuly, Doston S Uktamov, Sumayea Soni, Islomzhon Ikromzhon Ugli Atadzhanov

**Affiliations:** 1 Cardiovascular Medicine, Peoples' Friendship University of Russia, Moscow, RUS; 2 Cardiovascular Surgery, Peoples' Friendship University of Russia, Moscow, RUS; 3 Cardiology, I.M. Sechenov First Moscow State Medical University, Moscow, RUS; 4 General Medicine, Patrice Lumumba Peoples’ Friendship University of Russia, Moscow, RUS; 5 Nursing, Rajshahi Diabetic Association Nursing College, Rajshahi, BGD

**Keywords:** abdominal aortic aneurysm, endovascular aneurysm repair, enhanced recovery after surgery, infection prevention, perioperative care, risk stratification

## Abstract

Despite ongoing advancements in abdominal aortic aneurysm (AAA) repair, perioperative complications remain a major concern. This narrative review offers a novel analysis of emerging perioperative strategies, uniquely integrating recent guideline updates with evolving innovations such as AI-driven risk models, frailty and sarcopenia assessment, and personalized hemodynamic management. Unlike previous reviews, this article bridges evidence-based practices with real-world implementation challenges, highlighting barriers to guideline adherence, especially in low-resource settings.

The review evaluates the latest developments in anesthetic approaches, fluid and transfusion protocols, Enhanced Recovery After Surgery (ERAS) programs, and infection control measures, with a strong emphasis on tailored pathways for both open surgical repair (OPS) and endovascular aneurysm repair (EVAR). Long-term surveillance protocols and antimicrobial graft technologies are explored as key future directions. By connecting recent literature with practical, patient-centered applications, this work provides a forward-looking roadmap for optimizing AAA care across diverse clinical environments. Its novelty lies in presenting a comprehensive, multidisciplinary framework that incorporates precision medicine and implementation science to advance perioperative vascular surgery outcomes.

## Introduction and background

Perioperative optimization plays a critical role in abdominal aortic aneurysm (AAA) repair, directly impacting morbidity, mortality, and long-term patient outcomes. Given the high-risk nature of AAA repair, ensuring optimal preoperative risk assessment, intraoperative hemodynamic stability, and postoperative infection control is essential for reducing complications and improving recovery [1]. Advances in risk stratification models, blood pressure management protocols, and infection prevention strategies have significantly enhanced surgical outcomes in recent years [2].

Over the past decade, perioperative protocols have evolved to emphasize patient-specific approaches, integrating personalized risk assessment and enhanced recovery pathways. Traditional perioperative strategies focus on generalized protocols, but modern approaches now incorporate tailored management plans based on individual cardiac, pulmonary, and vascular risk factors [3]. This shift has been driven by evidence-based refinements in surgical guidelines, particularly European Society for Vascular Surgery (ESVS) and Society for Vascular Surgery (SVS) recommendations, which introduce new protocols for cardiac screening, blood pressure control, transfusion thresholds, infection prophylaxis, and improved survival rates [4,5]. This article aims to summarize the new perioperative recommendations for AAA repair, focusing on recent updates in cardiac and pulmonary risk assessment, intraoperative blood pressure management, transfusion protocols, and infection prevention strategies. In addition, the article explores how these perioperative strategies help reduce complications associated with both open surgical repair (OSR) and endovascular aneurysm repair (EVAR). By analyzing critical perioperative updates, the article will shed light on the latest advancements in AAA surgical management.

## Review

Methodology

This review was conducted as a narrative review rather than a systematic review to allow for a broader, thematic exploration of perioperative optimization strategies across multiple domains: cardiac, pulmonary, renal, and infectious complications, in both open and endovascular AAA repair. Given the diversity of study designs, variation in perioperative protocols, and evolving clinical guidelines, a narrative approach allowed for flexible integration of current evidence, expert consensus, and guideline-based practices that may not be directly comparable in quantitative synthesis.

A comprehensive search was performed using three major databases: PubMed, Web of Science, and Scopus, covering the period from 2014 to 2024. The aim was to identify relevant studies that address preoperative risk stratification, intraoperative management, enhanced recovery protocols, postoperative monitoring, and future innovations in AAA care. The search strategy combined Medical Subject Headings (MeSH) and free-text terms: (("Abdominal Aortic Aneurysm" AND (surgery OR repair)) OR ("Endovascular Aneurysm Repair" OR EVAR)) AND ("Perioperative Care" OR "Intraoperative Care" OR "Postoperative Care").

Two independent reviewers (Sidik AI and Al-Ariki MK) screened the titles and abstracts for relevance. Full-text review was then conducted to assess eligibility based on predefined inclusion and exclusion criteria. Discrepancies were resolved through discussion and consensus, with a third reviewer (Abdulmajid IS) consulted when necessary. Articles were included if they focused on the perioperative management of AAA and reported outcomes, protocols, or clinical recommendations relevant to modern surgical practice. Reference lists of selected articles were also searched. Studies that focused exclusively on basic science, animal models, device engineering, editorials, case reports, or conference abstracts were excluded. Table 1 outlines the criteria used for article inclusion and exclusion.

**Table 1:** Criteria for inclusion and exclusion of documents AAA, abdominal aortic aneurysm; OSR, open surgical repair; EVAR, endovascular aneurysm repair; RCT, randomized controlled trial

Criteria	Included	Excluded
Population	Adults undergoing AAA repair (OSR or EVAR)	Pediatric or non-aortic vascular surgery patients
Intervention/focus	Perioperative care (risk assessment, anesthesia, transfusion, infection control, etc.)	Basic science studies, device engineering, non-perioperative focus
Study types	Guidelines, systematic reviews, narrative reviews, RCTs, cohort and registry studies	Case reports, editorials, letters to the editor
Language	English	Non-English
Date range	2014-2024	Studies published before 2014

**Figure 1:**
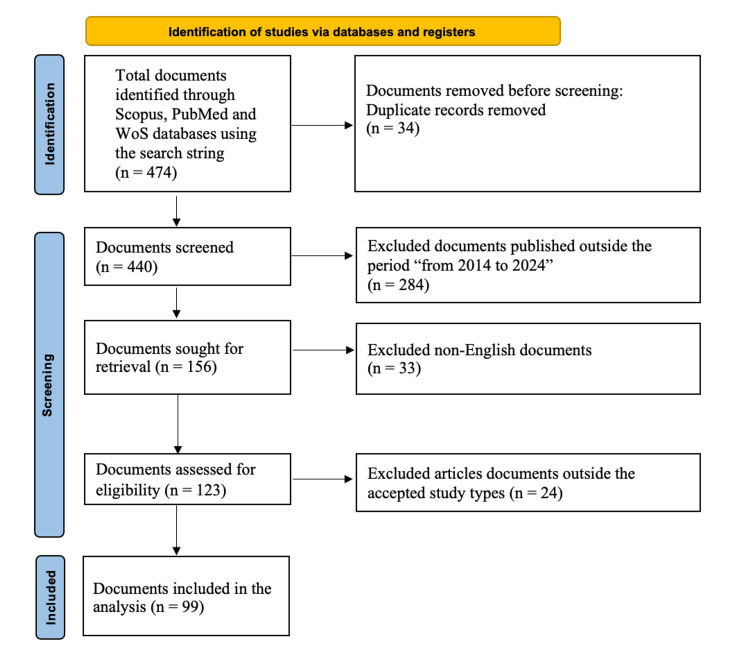
PRISMA flow diagram detailing steps in the identification and screening of sources PRISMA, Preferred Reporting Items for Systematic Reviews and Meta-Analyses

Following the review process using the PRISMA guidelines [6], a total of 474 potentially relevant studies were identified. After applying the inclusion and exclusion criteria, 99 references were used to support the manuscript’s thematic synthesis (Figure 1). These references span topics such as enhanced recovery protocols, hemodynamic monitoring, frailty assessment, and long-term surveillance after AAA repair. The findings were categorized into thematic domains and synthesized to highlight current best practices and future directions in perioperative AAA care. The structure and content of this review were guided by the Scale for the Assessment of Narrative Review Articles (SANRA) framework [7].

Preoperative optimization

Risk Stratification and Patient Selection

The success of AAA repair depends significantly on effective preoperative optimization, which includes risk stratification and patient selection.

Cardiac and pulmonary risk assessment: International guidelines recommend that comprehensive cardiac risk assessment should be performed for all patients undergoing AAA repair, as cardiac complications contribute to nearly 42% of perioperative deaths after non-cardiac surgery [5,8]. The 30-day risk of cardiovascular death or myocardial infarction is 5% or higher in OSR and 1-55% for EVAR [9]. Patients with active cardiovascular disease, such as unstable angina, decompensated heart failure, severe valvular disease, or significant dysrhythmias, require further specialist evaluation and optimization before proceeding with AAA repair. For patients without overt cardiac disease, the guidelines recommend evaluating clinical risk factors, functional capacity (metabolic equivalent (MET) score), and biomarkers [10]. A MET score of less than four (i.e., the patient is unable to climb two flights of stairs or jog a short distance) is associated with higher perioperative mortality and indicates the need for further cardiac workup [11]. Pulmonary function assessment is equally important, especially in smokers and patients with chronic obstructive pulmonary disease (COPD). Poor lung function is associated with higher rates of perioperative pneumonia and respiratory failure. Preoperative pulmonary function testing for high-risk patients and smoking cessation at least four to six weeks before surgery are necessary to improve lung function. Additionally, pulmonary rehabilitation programs, including incentive spirometry and bronchodilator therapy, are advised for patients with COPD. Screening for obstructive sleep apnea is also recommended in patients with obesity and chronic respiratory conditions to reduce postoperative pulmonary complications [12]. Table 2 shows risk factors for cardiovascular and pulmonary complications in patients undergoing repair for AAA.

**Table 2:** Key risk factors for cardiovascular and pulmonary complications following AAA repair FEV1, forced expiratory volume in 1 second; FVC, forced vital capacity; ASA, American Society of Anesthesiologists; AAA, abdominal aortic aneurysm; COPD, chronic obstructive pulmonary disease

Cardiac Risk Factors	Pulmonary Risk Factors
Advanced age	Age over 60 years
Prior symptomatic coronary artery disease	Existing COPD
History of heart failure	History of heart failure
Previous cerebrovascular events	Low serum albumin levels (≤3.5 g/dL)
Impaired kidney function (eGFR <60 mL/min or serum creatinine >170 µmol/L)	Reduced FEV1 (<70% of predicted)
Diabetes mellitus	Reduced FVC (<70% of predicted)
Reduced independence in daily living	FEV1/FVC ratio below 0.65
ASA physical status classification III or IV	-

Frailty and sarcopenia evaluation: Additionally, the assessment of frailty and sarcopenia has gained importance in surgical decision-making. Studies have demonstrated that frailty is linked to a five-fold increase in 30-day mortality risk after AAA repair, and low skeletal muscle mass (sarcopenia) predicts poor long-term survival. Despite this, more research is needed before these parameters are universally adopted into risk stratification models [13]. Based on current evidence, patient selection for elective AAA repair should be based on a combination of cardiovascular risk, functional capacity, and overall frailty assessment.

Prehabilitation and Lifestyle Optimization

Prehabilitation, a structured intervention to enhance patient fitness before surgery, has emerged as a promising strategy to reduce postoperative complications and enhance recovery [14].

Exercise interventions: A randomized controlled trial (RCT) from the UK demonstrated that supervised exercise training before AAA repair reduced cardiac, respiratory, and renal complications and shortened hospital stays. However, a Cochrane review highlighted that while prehabilitation may slightly reduce cardiac and renal complications, its impact on 30-day mortality and pulmonary complications remains uncertain [14].

Nutritional optimization: In addition, nutritional optimization is an often overlooked component of preoperative preparation. Hypoalbuminemia (<2.8 g/dL) has been associated with higher perioperative mortality, increased reoperation rates, and pulmonary complications following AAA repair. As a result, routine nutritional assessment before surgery, with dietary interventions and correction of deficiencies where necessary, is recommended [15].

Cardiovascular pharmacologic interventions: This plays a crucial role in perioperative risk reduction. Statins should be initiated at least four weeks before AAA repair and continued indefinitely postoperatively. Statins have been shown to reduce perioperative myocardial infarction and stroke risk, with meta-analyses confirming survival benefits in patients undergoing both open and endovascular repair [16]. Similarly, antiplatelet therapy with aspirin or clopidogrel is generally safe for patients undergoing AAA repair and does not significantly increase perioperative bleeding risk. However, dual antiplatelet therapy should be avoided unless the patient is at high cardiovascular risk [17].

Intraoperative considerations

Anesthetic Techniques

The choice of anesthesia for AAA repair remains a critical component of perioperative management, influencing postoperative recovery, morbidity, and mortality rates. There is an ongoing debate between general anesthesia (GA) and regional anesthesia (RA) techniques, particularly in the context of OSR and EVAR [18].

For EVAR, there is growing evidence supporting locoregional anesthesia (LRA) over GA. Studies suggest that LRA is associated with shorter procedure times, fewer ICU admissions, and lower 30-day mortality rates compared to GA [19]. The UK National Vascular Registry, which analyzed over 9,783 elective infrarenal EVAR procedures, found that RA was linked to a lower mortality rate compared to GA [20]. However, data remain inconclusive regarding anesthesia choice in AAA OSR, and individualized patient assessment remains the recommended approach [18].

A crucial development in perioperative care is the implementation of Enhanced Recovery After Surgery (ERAS) protocols, designed to minimize surgical stress and accelerate postoperative recovery. The integration of multimodal pain management, early ambulation, and nutrition optimization in AAA repair is encouraged. ERAS strategies, including epidural analgesia, opioid-sparing regimens, and early postoperative mobilization, have shown promise in reducing complications and hospital stay duration [21].

Abdominal Wall Closure and Mesh Reinforcement

**Figure 2:**
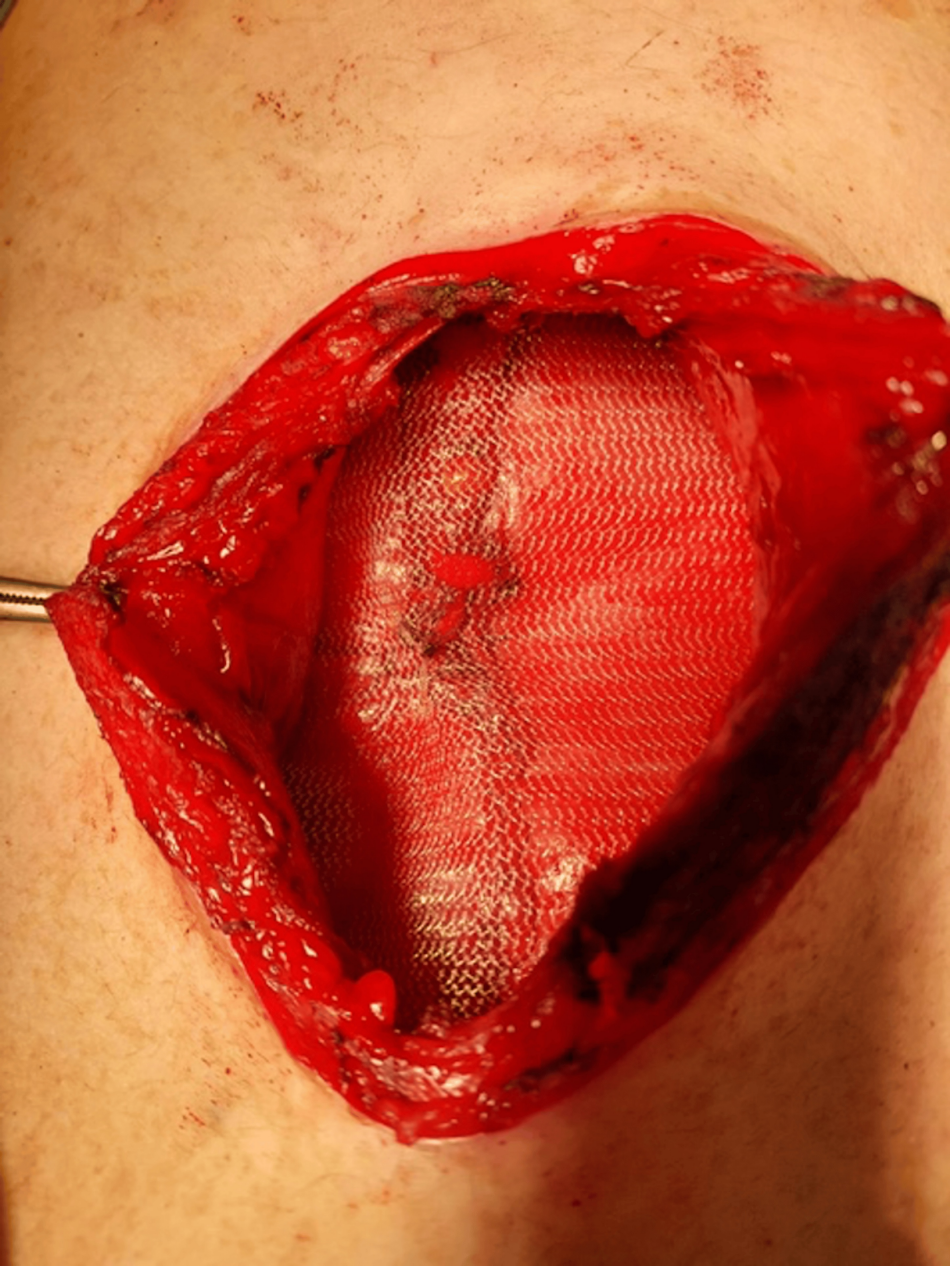
Prophylactic mesh reinforcement of abdominal wall following an open surgical repair for AAA OPS, open surgical repair; AAA, abdominal aortic aneurysm Photo credits: Authors

An often overlooked but critical intraoperative decision in open AAA repair is the method of abdominal wall closure. Recent high-level evidence from a meta-analysis of RCTs supports the routine use of prophylactic mesh reinforcement (Figure 2) over primary suture closure for midline laparotomy incisions. This approach significantly reduces the risk of incisional hernia (OR 0.20, 95% CI 0.09-0.43) and reoperation rates (OR 0.23, 95% CI 0.06-0.93), without increasing the risk of wound infection. Time-to-event analyses further confirmed a lower hazard of hernia formation in patients receiving mesh closure [22]. Given the high morbidity associated with incisional hernias and the lack of consensus in current guidelines, incorporating prophylactic mesh during the closure phase represents a practical, evidence-based strategy to improve surgical outcomes in open AAA repair.

Antibiotic Prophylaxis and Infection Control Strategies

Infection control remains a key priority in AAA repair, particularly in prosthetic graft implantation, where surgical site infections (SSIs) and graft-related infections can lead to catastrophic complications. For elective OSR, a single dose of cefazolin (or clindamycin for penicillin-allergic patients) is recommended 30-60 minutes before incision. In contrast, for EVAR procedures, routine antibiotic prophylaxis is no longer recommended in low-risk patients, but cefazolin or vancomycin should be considered for immunocompromised patients or prolonged procedures [23].

Additionally, new infection prevention measures include the use of chlorhexidine-alcohol for preoperative skin antisepsis, which has been shown to be superior to povidone-iodine in reducing SSIs. Preoperative decolonization strategies, such as nasal mupirocin and chlorhexidine body washes, are recommended in patients at high risk of methicillin-resistant Staphylococcus aureus (MRSA) colonization. Standardized intraoperative wound care protocols, including barrier dressings and antimicrobial sutures, are also emphasized to reduce postoperative infections [24].

To prevent prosthetic graft infections, guidelines recommend the use of antibiotic-coated grafts in high-risk patients and early identification of suspected graft infections, which may require long-term suppressive antibiotic therapy or, in severe cases, graft explantation and reconstruction. Routine postoperative surveillance, including imaging and biomarker-based infection screening, is advised in high-risk patients to detect and manage potential complications early [25].

Optimized Blood Pressure Control

Intraoperative blood pressure management plays a critical role in preventing AAA rupture, ischemia, and organ dysfunction. International guidelines introduced intraoperative blood pressure targets, emphasizing the maintenance of systolic blood pressure (SBP) between 100 mmHg and 120 mmHg during elective AAA repair. In cases of ruptured AAA, permissive hypotension strategies (SBP 70-90 mmHg) are recommended to reduce ongoing bleeding without compromising organ perfusion. Excessive blood pressure fluctuations should be avoided, as they increase the risk of myocardial infarction, stroke, and acute kidney injury [26,27].

Fluid Management and Transfusion Protocol

Effective intraoperative fluid management plays a vital role in preventing complications such as renal dysfunction and hypotension. The importance of goal-directed fluid therapy (GDFT) to maintain optimal perfusion pressure while avoiding fluid overload cannot be over-emphasized [28].

For elective AAA repair, restrictive fluid strategies are now favored over liberal resuscitation, as excessive intravenous fluid administration has been linked to pulmonary edema, increased ICU stays, and higher mortality rates [29]. Invasive hemodynamic monitoring tools, such as stroke volume variation and pulse pressure variation, are employed to guide intraoperative fluid administration [30]. Furthermore, international guidelines underscore the importance of maintaining adequate mean arterial pressure (MAP) during AAA repair [5,23]. Prolonged intraoperative hypotension (MAP <65 mmHg) has been associated with an increased risk of renal injury and myocardial infarction, necessitating the careful use of vasopressors and fluid resuscitation strategies [31].

New transfusion protocols favor restrictive transfusion strategies to prevent volume overload, hypercoagulability, and immune-mediated complications. The revised protocols recommend restricting transfusions unless hemoglobin levels fall below 7-8 g/dL in stable patients. Intraoperative blood conservation techniques, such as cell salvage and autologous transfusion, reduce allogeneic blood transfusion requirements [32,33]. These measures align perioperative fluid and transfusion strategies with modern evidence-based practices, ultimately reducing the risk of organ dysfunction, surgical site complications, and thrombotic events.

Perioperative Anticoagulation and Blood Management

Anticoagulation during AAA repair is critical to prevent thromboembolic complications while minimizing bleeding risks. Systemic heparin should be administered intraoperatively for both OSR and EVAR, with doses ranging between 50 IU/kg and 100 IU/kg [34]. However, recent studies suggest that a weight-based dosing strategy with activated clotting time monitoring may be more effective in optimizing anticoagulation while reducing excessive bleeding risks [35].

In terms of antiplatelet therapy, the guidelines reaffirm that aspirin or clopidogrel monotherapy is generally safe and does not significantly increase perioperative bleeding risks. However, patients on dual antiplatelet therapy, such as those with recent stent placement, require individualized risk-benefit assessment [17]. Warfarin should be discontinued at least five days preoperatively and direct oral anticoagulants at least two days before surgery to reduce bleeding risks [23].

Postoperative care and complication prevention

Postoperative Monitoring and ICU Management

Postoperative care plays a critical role in ensuring optimal recovery and minimizing complications after AAA repair. All patients undergoing OSR and high-risk individuals undergoing EVAR should be monitored in an ICU or high-dependency unit in the immediate postoperative period. This is particularly important for patients with pre-existing cardiovascular disease, renal dysfunction, or respiratory compromise, as they face a higher risk of perioperative complications [5,23].

Early postoperative monitoring involves continuous hemodynamic assessment, oxygenation optimization, and early detection of graft-related complications such as endoleaks or graft thrombosis. Patients who undergo OSR often require more intensive monitoring due to the greater physiological stress of the procedure, whereas EVAR patients may benefit from a more rapid recovery pathway with earlier discharge if they remain hemodynamically stable [5,23].

The adoption of ERAS protocols has significantly improved postoperative outcomes in vascular surgery. Pain control strategies include RA techniques, epidural analgesia, and non-opioid analgesics, all of which contribute to improved respiratory function and reduced risk of deep vein thrombosis [21].

Prevention of Major Complications

Despite advancements in surgical techniques and perioperative care, postoperative complications remain a significant concern in AAA repair. Renal dysfunction, particularly acute kidney injury, is a frequent complication after AAA repair, especially in patients undergoing OSR [36]. To minimize this risk, GDFT, nephroprotective strategies, and the avoidance of nephrotoxic medications such as NSAIDs are recommended. Adequate renal perfusion should be maintained intraoperatively, and efforts should be made to reduce prolonged hypotension, which is strongly linked to postoperative acute kidney injury [37,38].

Pulmonary complications, including atelectasis, pneumonia, and respiratory failure, are also common after AAA repair. Preventive measures include early ambulation, pulmonary physiotherapy, and preoperative smoking cessation. Studies show that patients who undergo early mobilization within 24 hours post-surgery have significantly lower rates of pulmonary complications and ICU readmission [39,40].

Wound infections remain a concern, particularly in open AAA repair, where large surgical incisions increase the risk of SSI. A comprehensive infection prevention strategy, including perioperative antibiotic prophylaxis, meticulous surgical technique, and glycemic control in diabetic patients, is necessary to mitigate such complications. For patients at high risk of graft infections, the guidelines suggest long-term antibiotic suppression therapy [23,24].

Additionally, enhanced surveillance protocols are essential for the early detection of graft-related complications, such as endoleaks, graft thrombosis, and anastomotic complications. Routine postoperative imaging with computed tomography angiography (CTA) or duplex ultrasound (DUS) to monitor graft integrity and detect any potential issues before they become life-threatening are also recommended [41,42].

Long-Term Outcomes and Follow-Up

Long-term survival after AAA repair is influenced by multiple factors, including age, sex, baseline comorbidities, and post-repair surveillance adherence. Post-repair surveillance protocol for both OSR and EVAR to detect late complications and ensure optimal long-term outcomes should be implemented in vascular centers [23].

For post-EVAR surveillance, CTA should be performed within 30 days postoperatively, followed by annual CTA or DUS in high-risk patients to monitor for endoleaks, graft migration, and sac enlargement. Given the risk of late complications with EVAR, long-term imaging is crucial to ensure the durability of the repair and prevent rupture due to undetected endoleaks [41,43].

For post-OSR surveillance, imaging follow-up is recommended every five years, as complications such as anastomotic aneurysms or new aortic pathology tend to develop much later than in EVAR patients. However, if patients exhibit new symptoms or signs of vascular compromise, more frequent imaging may be warranted [44].

Beyond surveillance, long-term cardiovascular risk management plays a pivotal role in improving survival rates after AAA repair. Patients should receive comprehensive secondary prevention measures, including lifelong statin therapy, antiplatelet agents, and strict blood pressure control. Evidence indicates that patients who adhere to these risk reduction strategies experience lower rates of major cardiovascular events, including myocardial infarction and stroke [5,23].

Future directions in perioperative AAA management

Personalizing Perioperative Protocols for High-Risk Patients

With the increasing adoption of precision medicine, there is a growing need for individualized perioperative protocols tailored to each patient's cardiovascular, respiratory, and metabolic risk factors. The integration of AI-driven predictive models, such as the Vascular Study Group of New England (VSGNE) AAA mortality risk prediction model, can enhance risk stratification and surgical planning, thereby ensuring optimal perioperative management for high-risk patients [5,23,45].

The VASCUL-AID project, a European multi-center initiative, is currently evaluating AI-based predictors for AAA progression, with the aim of refining risk prediction and optimizing treatment planning [46,47].

AI-assisted risk assessment models can analyze large datasets of patient characteristics, surgical outcomes, and intraoperative variables to develop personalized perioperative protocols [45]. For example, AI can predict which patients may require stricter hemodynamic control or specific transfusion strategies based on their unique physiology. This could allow for tailored blood pressure targets, optimizing perfusion while reducing ischemic complications in both open and endovascular AAA repair. Additionally, AI-driven decision-support tools could assist surgeons in selecting the most effective fluid management, ventilation strategies, and perioperative medications, ensuring safer, patient-specific surgical care [48,49]. 

Innersight Labs, a UK-based company, has developed an AI-enhanced platform that combines 3D reconstruction and holographic visualization to improve training and planning for AAA procedures. Their technology creates patient-specific holograms of aneurysms, which can be used for surgical training, multidisciplinary team (MDT) simulations, and preoperative planning. In particular, it supports EVAR by enabling precise measurement of key anatomical features such as neck length, diameter, and angulation. The holographic models can be viewed through VR headsets or projected directly into the operating room, offering surgeons real-time guidance and enhancing procedural accuracy [50].

Long-Term Surveillance and Infection Prevention Strategies

Ensuring long-term graft durability and preventing late complications are key areas for future research in AAA management. Postoperative surveillance protocols need to be optimized to detect early graft-related complications, infection risks, and aneurysm progression [41,51].

One of the most pressing concerns in vascular surgery is the risk of prosthetic graft infection. Despite advances in antibiotic prophylaxis and infection control strategies, late graft infections remain a serious, life-threatening complication. Research into novel biomaterials with antimicrobial properties may help develop infection-resistant vascular grafts, reducing the need for long-term antibiotic therapy and revision surgeries [23,25].

Another critical aspect of postoperative management is refining long-term imaging surveillance protocols. AI-assisted imaging analysis could help identify subtle structural changes in vascular grafts, allowing for earlier intervention in cases of graft stenosis, migration, or endoleaks [48]. Future studies should focus on optimizing the frequency and modality of follow-up imaging, ensuring that patients receive timely and cost-effective surveillance without unnecessary radiation exposure.

The Impact of Minimally Invasive Techniques on Perioperative Outcomes

Minimally invasive approaches, particularly EVAR, continue to evolve, with newer techniques aiming to reduce procedural risks, improve durability, and enhance long-term outcomes. There is a growing role of complex endovascular solutions, including fenestrated and branched EVAR, which allow the treatment of more anatomically challenging aneurysms [52].

Despite these advancements, concerns regarding the long-term durability of EVAR remain, with studies showing higher reintervention rates compared to OSR. The development of new low-profile stent grafts, polymer-sealing technologies, and bioresorbable scaffolds holds promise for improving endovascular durability and reducing post-procedural complications [53,54].

Another major advancement is radiation-free endovascular navigation systems, such as fiber optic real-shape and electromagnetic tracking, which may significantly reduce radiation exposure for both patients and surgical teams. These technologies are still in the early stages, but their adoption could enhance procedural safety and broaden the applicability of minimally invasive AAA repair [55].

Research Gaps and Areas Requiring Further Investigation

Despite the progress in perioperative AAA management, several research gaps remain. One critical area is the lack of high-quality evidence for optimizing patient selection criteria for AAA repair. While the 55 mm threshold for elective AAA repair in men is widely accepted, data supporting this threshold remain weak. Similarly, there is no consensus on the optimal repair threshold for women, with ongoing trials such as the Women’s Ischemia Trial to Reduce Events In Non-Obstructive CAD (WARRIORS) trial investigating whether women might benefit from earlier EVAR intervention [56].

The role of pharmacological interventions in AAA progression remains another area requiring further study. Metformin, an antidiabetic drug, has emerged as a promising candidate for slowing aneurysm growth, with multiple ongoing RCTs evaluating its effectiveness. It may stabilize AAAs by reducing inflammation, inhibiting matrix degradation, preserving vascular smooth muscle cells, and improving endothelial function. It activates AMPK and downregulates matrix metalloproteinases (MMPs) and pro-inflammatory cytokines [57,58]. Additionally, drug-coated balloons and localized drug delivery via EVAR devices are in the early stages of development and could significantly alter future treatment strategies [59].

Furthermore, long-term follow-up strategies for post-EVAR patients remain inconsistent, with the optimal frequency of imaging surveillance beyond five years still unclear. The guidelines highlight the need for large, high-quality registry data to determine the most effective follow-up schedules and early intervention protocols for high-risk patients [23].

Barriers to Implementation of AAA Guidelines in Real-World Settings

Despite the comprehensiveness of the ESVS and SVS guidelines, several barriers hinder their consistent implementation in real-world settings. These include infrastructural and resource limitations, especially in low-volume or resource-constrained centers, and a lack of interdisciplinary coordination required for comprehensive perioperative optimization. Additionally, the high number of recommendations, many based on Level C evidence, combined with the limited integration of AI or decision-support systems, further complicates clinical application. Financial constraints and variations in reimbursement policies may restrict the adoption of advanced devices and surveillance protocols. Finally, patient-level factors such as comorbidities, adherence challenges, and social determinants of health must be addressed to ensure the effective translation of guidelines into practice.

Limitations of Current Guidelines

While current international guidelines represent a significant advancement, several limitations remain. Many recommendations still rely on low-level evidence or expert consensus, particularly in areas such as pre-habilitation, frailty assessment, and EVAR follow-up. There remains a lack of gender-specific thresholds for repair and a clear framework for incorporating emerging technologies like AI into perioperative planning. These gaps underscore the need for further research and continual refinement of AAA surgical guidelines to reflect personalized, data-driven approaches.

## Conclusions

Recent advances in perioperative care have significantly improved outcomes in AAA repair. Key strategies include enhanced cardiac and pulmonary risk assessment, tailored hemodynamic and transfusion protocols, and evidence-based infection control. ERAS protocols and early mobilization have reduced complications and hospital stays. Emerging technologies such as AI-driven risk models and antimicrobial grafts hold promise for further improving care. However, gaps remain in long-term surveillance, gender-specific thresholds, and implementation across varied clinical settings. A multidisciplinary, patient-centered approach remains essential to optimizing outcomes and advancing the standard of care in both open and endovascular AAA repair.
